# Characterization and Quantification of Selenoprotein P: Challenges to Mass Spectrometry

**DOI:** 10.3390/ijms22126283

**Published:** 2021-06-11

**Authors:** Jérémy Lamarche, Luisa Ronga, Joanna Szpunar, Ryszard Lobinski

**Affiliations:** 1 IPREM UMR5254, E2S UPPA, Institut des Sciences Analytiques et de Physico-Chimie Pour l’Environnement et les Matériaux, CNRS, Université de Pau et des Pays de l’Adour, Hélioparc, 64053 Pau, France; luisa.ronga@univ-pau.fr (L.R.); joanna.szpunar@univ-pau.fr (J.S.); ryszard.lobinski@univ-pau.fr (R.L.); 2 World-Class Research Center “Digital Biodesign and Personalized Healthcare”, IM Sechenov First Moscow State Medical University (Sechenov University), 119435 Moscow, Russia; 3 Chair of Analytical Chemistry, Warsaw University of Technology, Noakowskiego 3, 00-664 Warsaw, Poland

**Keywords:** selenoprotein P, mass spectrometry, metrology, selenium, cancer, biomarker, selenocysteine

## Abstract

Selenoprotein P (SELENOP) is an emerging marker of the nutritional status of selenium and of various diseases, however, its chemical characteristics still need to be investigated and methods for its accurate quantitation improved. SELENOP is unique among selenoproteins, as it contains multiple genetically encoded SeCys residues, whereas all the other characterized selenoproteins contain just one. SELENOP occurs in the form of multiple isoforms, truncated species and post-translationally modified variants which are relatively poorly characterized. The accurate quantification of SELENOP is contingent on the availability of specific primary standards and reference methods. Before recombinant SELENOP becomes available to be used as a primary standard, careful investigation of the characteristics of the SELENOP measured by electrospray MS and strict control of the recoveries at the various steps of the analytical procedures are strongly recommended. This review critically discusses the state-of-the-art of analytical approaches to the characterization and quantification of SELENOP. While immunoassays remain the standard for the determination of human and animal health status, because of their speed and simplicity, mass spectrometry techniques offer many attractive and complementary features that are highlighted and critically evaluated.

## 1. Introduction

Selenium, originally known for its toxicity, was subsequently recognized as an essential trace element [1] and has been increasingly marketed as a life-style drug [2]. The importance of Se is related to its antioxidant properties, its role in the endocrine and immune system, as well as its involvement in the protection against certain diseases, such as cancer, diabetes, cardiovascular or immune system disorders [3]. The narrow range of the optimum selenium concentration in biological fluids, the multiplicity of its chemical forms and the diversity of its biological activities are driving interest for precise molecular markers of its status in various organisms. 

The activity of Se is mainly mediated by selenoproteins. They contain in their sequence at least one selenocysteine (SeCys, U), which is genetically encoded and often referred to as the 21st amino-acid [4]. In the presence of a specific stemloop RNA sequence element (SECIS), the UGA codon, normally acting as a stop codon to terminate translation, codes for the insertion of SeCys into a polypeptide chain [2,5]. The pioneering works of Gladyshev group [6,7] started the development of algorithms predicting the existence of many selenoproteins in different biological species [8,9]. The number of selenoproteins that can be expressed in different organisms varies, ranging from 1 in certain insects, to 9 in common spider, 25 in humans [10], and 32 in oysters [11]. In vertebrates, the mRNA encoding SELENOP is distinct from that encoding other selenoproteins, in that it contains two SECIS elements instead of one [12].

**Figure 1:**
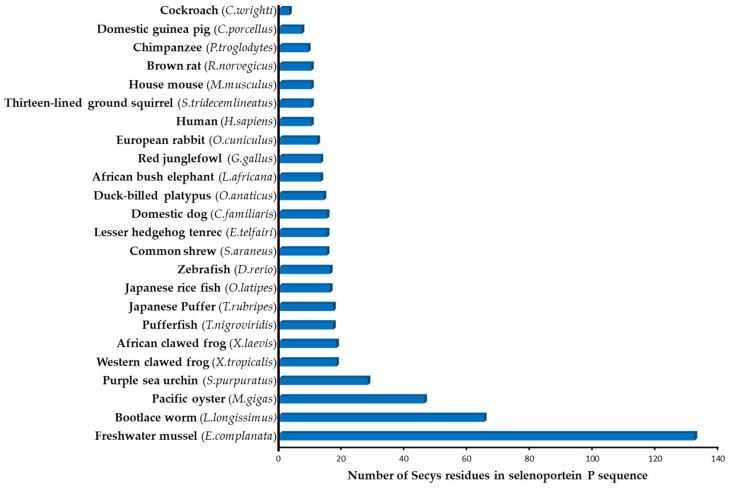
Putative number of SeCys residues in SELENOP in different organisms (according to [11,12,14]).

Selenoprotein P is unique amongst all the characterized selenoproteins in that it contains multiple SeCys residues, whereas all other selenoproteins contain only one SeCys. The number of SeCys residues in SELENOP from different organisms varies (Figure 1), amounting to 10 for humans, 46 in oysters [11] and 132 in one-species bivalves [13]. 

SELENOP was first reported in 1973 by Burk et al. [15] and Rotruck et al. [16]. McConnell et al. [17] and Motsenbocker et al. [18] found that SELENOP was synthetized in the liver of rats and secreted into the plasma. A few years later, the existence of SELENOP was confirmed in humans, where it is considered as a valuable marker for nutritional selenium status [19]. In 2016, Gladyshev et al. proposed a non-ambiguous notation for all selenoproteins using the root symbol SELENO followed by a letter, leading to the creation of the abbreviation SELENOP for selenoprotein P, replacing the previously used as abbreviations such as SeP, SEEP1, SelP [20]. Expression, functions and role of SELENOP in mammals were reviewed [21]. 

As a biomarker, SELENOP can discriminate between the specific and nonspecific (and therefore non-significant) incorporation of Se in proteins [22,23]. It responds to Se supplementation over a wider range of intakes than GPx3 [24]. In marginally supplied individuals, low serum Se was found to be mirrored by the circulating SELENOP concentration, but not by the GPx3 activity [25]. In studies of populations with relatively low selenium intakes, SELENOP was found to respond to different dietary selenium forms [26]. Serum SELENOP concentration can be more than a biomarker of Se status: it was proposed to be used for the diagnosis and assessment of treatment efficacy and long-term prognosis in patients with pulmonary arterial hypertension [27] and hypertension [28]. Recently, the mortality risk from COVID-19 was shown to be associated with selenium deficiency, and more specifically with SELENOP deficiency, causing a dysregulation of the redox homeostasis in pathological conditions which resulted in an excessive reactive oxygen species (ROS) generation [29,30].

During the last 40 years, many functions have been attributed to SELENOP including its involvement in the storage of selenium in the brain, testis [23,31] and kidney [32], defence against oxidative stress [33], loss of fertility [23], polycystic ovary syndrome [34] or regulation of heavy metals concentration [35,36]. SELENOP was reported to play a role in the potential development of various forms of cancer [37,38,39,40] and to be associated with neurodegenerative diseases such as Alzheimer’s [39,41,42,43]. SELENOP was evoked as a therapeutic target for type 2 diabetes [41,44,45,46] because of its role in the regulation of glucose metabolism and insulin sensitivity. Recent advances in the understanding of the role of SELENOP and its potential medical/pharmaceutical implications were reviewed [47].

In order to fully elucidate the mode of action and the importance of SELENOP in the different pathological pathways, a complete structural characterization, quantitative determination and localization of SELENOP in the tissues are needed [48]. Mass spectrometry techniques are uniquely placed to address these ongoing challenges. The goal of this review is to critically discuss the current state of-the-art analytical methods for the characterization and determination of SELENOP, identify the open questions and indicate how they can be answered.

## 2. Selenoprotein P Characteristics

To date, the three-dimensional structure of SELENOP has not been solved. A possible reason for this is the difficulty in the exogenous over-expression of SELENOP in bacteria or in cultured cells, because of the presence of multiple Sec residues in the polypeptide [47]. Studies using recombinant SELENOPP have been rare [49]. Consequently, the SELENOP characterization was carried out on protein expressed endogenously by cell lines or purified from plasma and serum.

**Figure 2:**
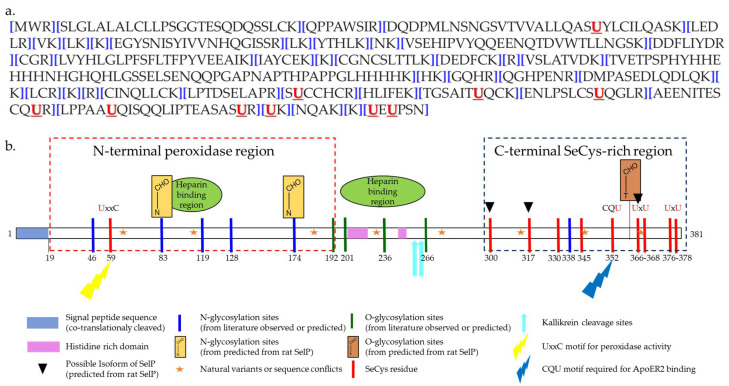
Sequence of human SELENOP. (**a**) Amino-acid sequence of human SELENOP [53]; (**b**) schematic representation of human SELENOP (on the basis of [47,53] and Uniprot database).

The matured predominant isoform of human SELENOP consists of 359 amino acids (AA) after cleavage of the predicted signal peptide (AA 1–21) [47] (Figure 2a). The selenium content (as SeCys) of SELENOP is distributed into two parts [21,47]. The N-terminal domain contains one selenocysteine at the 40th amino-acid in a U-x-x-C redox motif. The shorter C-terminal domain contains multiples selenocysteine, up to 9 in total for rats, mice, and humans [50,51] The N-terminal domain is responsible for the enzymatic activity of this protein, while the C-terminal domain acts as the Se supplier [52] (Figure 2b).

SELENOP is not a homogenous protein. As a consequence of the SeCys gene expression by stop codon recoding, multiple forms of the SELENOP of different molecular weight exist in terms of relation to genotype, differential splicing, premature interrupted translation at one of the UGA codons, limited post-translational proteolysis or partial replacement of SeCys by Cys [47,54,55,56,57]. To date, four isoforms have been identified in rat plasma. Beside the full-length isoform with 10 selenocysteine residues, shorter isoforms terminating at the 2nd, 3rd, and 7th selenocysteine [56], with 1, 2 and 6 selenocysteines, respectively, were reported [58]. Interestingly, in a study involving ca. 2000 subjects, the average determined number of Se atoms per SELENOP molecule (5.4) was considerably below the predicted number of 10 Se atoms [59].

In the native form, SELENOP contains selenenylsulfide and disulfide bridges. It possesses three N-glycosylation sites at the *N*-terminus and one *O*-glycosylation site at the C-*termin*us [60] and thus, can be referred to as glycoprotein. One highly glycosylated form is secreted by the liver [61]. Selenium-supplemented HepG2 hepatoma cells secrete N-glycosylated SELENOP as well [62]. The post-translational modifications are thought to confer a particular structural behavior to SELENOP and to protect selenium by reducing its reactivity [10].

## 3. Isolation of Selenoprotein P from Biological Samples Prior to Mass Spectrometry (MS) Characterization

SELENOP is typically present in serum at a level of ca. 50 ng/mL. Its concentrations in cultured cells, breast milk, or tissues are an order of magnitude lower. The abundance of SELENOP in human serum exceeds that of plasma glutathione peroxidase (GPx) and selenized albumin (SeAlb) [31]. 

Taking into account the presence of a multitude of proteins in a 1000-fold excess, any characterization of SELENOP by mass spectrometry should be preceded by its isolation and enrichment. The methods are usually based on the immunoaffinity precipitation or chromatography (mono- and/or poly-clonal antibodies) [52,58,63,64,65,66] or chemical affinity (heparine [56,67,68] or immobilized transition metals [49,69]).

### 3.1. Immunoaffinity Precipitation and Chromatography 

The use of immunoaffinity has been largely explored for isolation of SELENOP, but this strategy is critically dependent on the quality and selectivity of antibodies. The cross-activity of one antibody with an antigen from different species is not granted and must be tested. Moreover, the activity of the antibody is highly variable for the serum of one animal to the other and must also be systematically tested. 

The first purification of SELENOP by immunoprecipitation was performed from rat serum, using monoclonal antibodies [63]. Subsequently, Akesson et al. [58] used the rat monoclonal antibodies for purification of human SELENOP. Other groups reported the preparation [64,70] or use [71] of monoclonal antibody raised against rat SELENOP [64]. No cross-reactivity with plasma from five animal species [58] was observed. Commercially available antibodies against murine SELENOP do not cross react with the human orthologue [69]. Recombinant rat SELENOP was efficiently immunoprecipitated by a commercial penta-histidine antibody but not by the tetrahistidine one [49]. 

Considerable developments in the field of SELENOP antibodies have taken place in the recent years driven by the need for the development of ELISA kits (see below). They are based on the use of antibodies prepared by using recombinant mutant SELENOP as immunogen. These expressed SELENOP mutants are characterized by the absence of SeCys which are all replaced by Cys [72] or Ser [73]. A recombinant SELENOP commonly used as immunogen for commercial antibody development encompasses the 60-299 SELENOP sequence without SeCys residues [74]. Alternatively, SELENOP purified from human serum was employed as immunogen [52,70,75].

The antibody-based methods for purification and measurement of SELENOP may not allow the distinction among the isoforms [56]. Indeed, antibodies are usually directed towards one of its domains (N or C). Consequently, they capture not only full-length SELENOP but also its N- or C-terminal side domain fragments. In vivo, SELENOP is cleaved by plasma kallikrein which generates N-terminal and C-terminal fragments of SELENOP [75]. The combined use of antibodies specific for N- or C-terminal SELENOP side domain fragments allows the differentiation between the full-length SELENOP from total SELENOP (truncated and full length) [52,54,66]. 

Technically speaking, immunoprecipitation of SELENOP can be conveniently performed with commercial antibodies conjugated to polystyrene superparamagnetic beads [65]. GPx3 is co-immunoprecipitated with SELENOP, resulting in incomplete separations which is likely to be due to the similarity in the structure of the N-terminal part of SELENOP and GPx3 [65]. This point has not been addressed by recent studies where combined antibodies specific for the different SELENOP *termini* were employed.

### 3.2. Heparin Affinity Methods

Two histidine-rich stretches containing up to 10 sequential basic amino acids are present in the 185–198 and 225–234 amino-acid sequences in rats [67]. The rat and human SELENOP sequences encode two His-rich regions: the first region consists of 8 (rat) or 9 (human) histidines out of 14 residues, and the second, a stretch of 7 (rat) or 4 (human) consecutive histidines [76]. The presence of these stretches confers to SELENOP a feature of binding to heparin [67].

SELENOP binds to heparin as a function of pH. The binding is facilitated by an increase in protonation of histidine residues. Therefore, SELENOP will bind to heparin under acidic conditions but remains unbound at physiological pH [21]. The pKa of histidine (7.0) explains the release of SELENOP from heparin at alkaline conditions [67].

Heitland et al. were able to isolate SELENOP using heparin column from other serum proteins with a total recovery of selenium (96%) [68]. A recovery above 90% was reported [77]. Problems linked to nonspecific adsorption of plasma-extracellular glutathione peroxidase (GPx) and albumin on the heparin affinity column were evoked [77]. 

Purified SELENOP can be separated in three peaks using heparin chromatography suggesting its capability to discriminate amongst the isoforms [56].

### 3.3. Immobilized Metal Affinity Methods (IMAC)

These histidine-rich regions in conjunction with the Cys and SeCys content, are likely responsible for the coordination to heavy metals such as, e.g., mercury [76]. The presence of such motifs makes it possible to retain SELENOP on an IMAC-sepharose column loaded with cobalt [69]. Co^2+^ was found superior to Cu^2+^, Ni^2+^, Zn^2+^, and Cd^2+^ for metal affinity LC [69].

The Ni-agarose chromatography was performed using Ni-NTA spin columns and turned out to be efficient for the isolation of SELENOP, prior to SDS PAGE and Western blotting analysis [49].

### 3.4. Sequential Purifications

In order to increase the purity of the isolated SELENOP, the above discussed steps can be employed in a sequence. Akesson et al. reached a 1000-fold purification of SELENOP by combining immunoaffinity LC and heparin [58]. The immunoaffinity purified protein was further separated into several forms using heparin-sepharose column [64]. 

Daegen et al. separated plasma into three components (GPx, SELENOP, and Alb) using heparin-sepharose and blue 2-sepharose (to remove SeAlb) [78]. A combination of IMAC and heparin offered a 15,000-fold enrichment of SELENOP [69]. Isolation of electrophoretically pure SELENOP was reported to be achieved in three steps: heparin agarose, ultrafiltration concentration, anion-exchange, Ni-NTA-agarose [61]. The combination of heparin-sepharose CL-GB, Q Sepharose F and Ni-NTA agarose chromatography, followed by desalting by gel filtration, allowed a 13,000-fold purification of SELENOP with an overall yield of 16% [79]. The low yield of sequential purification is often due to the multiplication of steps inducing loss of protein. In recent studies, the use of heparin was shown to be the most efficient for SELENOP purification with a yield of 96% [68], however this method does not concentrate the protein.

## 4. Detection and Characterization of SELENOP by Soft Ionization Mass Spectrometry

The isolated SELENOP can be formally identified without mass spectrometry by the N-terminal amino-acid sequence (or microsequencing) [79]. However, the advantage of MS in terms of sensitivity and speed cannot be overestimated. So far, to our knowledge, there have been no mass spectra published for the intact full-length SELENOP and their truncated isoforms. Most of the published MS data concern the analysis of peptides, obtained after tryptic digestion, by matrix-assisted laser desorption ionization (MALDI) or electrospray ionization MS. The latter allows the determination of the peptide sequence upon collision induced fragmentation (MS/MS). The list of the reported peptides allowing the 100% sequence specificity and they correspondence to full-length or truncated isoforms is given in (Table 1).

A number of bioinformatic tools have been developed for the detection of selenoproteins in high throughput MS schemes [9,80]. Several enzymes (such as trypsin or endoproteinase) can be used to lyse the proteins and to obtain different SELENOP characteristic peptides. The inconvenience of bottom-up proteomics approaches is that it is not always possible to identify isoforms from which the peptides are derived. Their advantage is the capability to deal with the post-translational modification.

The major challenges in mass spectrometry analysis include the ability to be able to distinguish between isoforms, to complete the characterization of truncated forms and to develop analytical methods for their quantification. The potential of top-down proteomics for this purpose is very promising [81].

### 4.1. MALDI-MS

MALDI-MS allowed the discovery in rat plasma of three SELENOP isoforms that have identical N-*termini*, and differ in the length of the amino-acid chain [56]. The full-length SELENOP and the isoforms were separated by SDS PAGE. They were reduced, alkylated, deglycosylated and digested with trypsin. Additional peptides could be identified by digestion with Glu-C of the SelP isolated by heparin, without the need for its subsequent purification by SDS PAGE. Mass spectrometry could identify the C-*termini* of the isoforms according to the prediction (at the 2nd, 3rd and 7th selenocysteine residue) [56].

MALDI-MS was also used to identify the sites of glycosylation of the full-length SELENOP [60]. The procedure was based on a treatment with PNGase F, which cleaves off asparagine-linked carbohydrates and converts the residue asparagine to aspartic acid. Of the five potential glycosylation sites, three located at residues 64, 155 and 169 were occupied, and two at residues 351 and 356 were not occupied. Threonine 346 was variably O-glycosylated. Full-length SELENOP was found to be both N- and O-glycosylated [60].

MALDI-MS was also essential in the identification of Se-S and disulfide linkage sites [60]. The strategy of sample preparation for the determination of the oxidation state of the cysteine residues consisted of the alkylation of all free cysteines and selenocysteines in the short form of SELENOP with iodoacetamide, digestion with endoproteinase, and deglycosylation [60]. A selenide-sulfide bond was found in the shortened isoform to be analogous to the selenol-thiol pair considered to be redox-active [60]. Two selenylsulfide bonds were identified by MALDI-MS in a peptide isolated from a tryptic digest of rat SELENOP [82].

### 4.2. Electrospray MS

The basic advantage of electrospray ionization (ESI) MS over MALDI is the possibility to sequence peptides in LC-MS/MS on the basis of the *m*/*z* of their *b* and *y* fragments. ESI MS was used to verify the sequences of the putative glycosylated peptides in rat SELENOP [60] and the confirmation of the existence of disulphide linkages [60,82]. 

High pressure liquid chromatography (HPLC)-MS/MS was a convenient technique for the identification of SELENOPeptides in a tryptic digest of purified SELENOP. It allowed the formal confirmation of SELENOP presence in human breast milk [83]. A sequence with a coverage of 80% of the theoretical one was reported on the basis of the tryptic digest analysis, and two SELENOPeptides were formally identified [83]. 

SELENOP, being a low-abundant protein in serum, was not detected by a regular shotgun proteomics approach [84]. The analysis of the fraction purified by heparin allowed the detection of three unique SELENOP peptides identified by only one post-translational modification for each, and a sequence coverage of 41.5% [84]. The purification of SELENOP by SDS PAGE and blotting, which was followed by tryptic digestion and HPLC-MS/MS, allowed the identification of 7 SelP peptides totalling 115 post-translational modifications (none of which contained selenium) in the 49 kdaSELENOP band in the blot, accounting for a sequence coverage factor of 17.7%. Selenium contained peptides missed by the regular shotgun proteomics procedure. The identification of two SELENOPeptides increased the sequence coverage to 24.4% [84]. Three isoforms of SELENOP were identified by this method [65]. 

Human SELP still needs a complete MS characterization covering all the SELENOPeptides. The recent data obtained are summarized in Table 1. 

The developments in soft ionization mass spectrometry open new perspectives in the detection of SELENOP in broad scope proteomic studies. The method is based on nano-flow liquid chromatography (LC) with electrospray MS/MS detection and data-independent acquisition MS [85]. For instance, label-free proteomics showed selenoprotein P as the most abundant proteins in milk of cows, more precisely, in cows producing milk with A2A2-β-casein variants [86]. The protocols can be quantitative by using isobaric tagging for relative and absolute Quantification (iTRAQ) [80,87,88,89]. 

**Table 1:** List of SELENOPeptides used for the SELENOP mass spectrometry identification on the basis of a partial sequence (peptides with the same sequence are highlighted in colors).

Matrix	SELENOP Specific Sequence with Sec Detected	Identified Isoform	Ref.
Rat plasma	^28^GTVTVVALLQASUYLCLLQASRLE^51^ ^239^QGHLESUDMGASEGLQLSLAQR^260^ ^252^GLQLSLAQRKLURRGCINQLLCKLSEE^278^ ^298^SGSAITUQCAENLPSLCSUQGLFAEEK^324^ ^333^SPPAAUHSQHVSPTEASPNUSUNNK^357^ ^348^ ASPNUSUNNKTKKUKUNLN^366^	4 Isoforms: 50 kDa (full length) 49 kDa (terminated at 351) 38 kDa (terminated at 262) 36 kDa (terminated at 244)	[56]
Rat plasma	^28^GTVTVVALLQASUYLCLLQASR^49^ ^328^SCQCRSPPAAUHSQHVSPTE^347^	1 Isoform terminated at 244	[60]
Rat plasma	^298^ SGSAITUQCAENLPSLCSUQGLFAEEK^324^	*n.d.*	[82]
Human plasma	^322^ ENLPSLCUQGLR^334^ ^335^ AEENITESCQUR^346^	*n.d.*	[84]
Human plasma	^312^ TGSAITUQCK^321^ ^322^ ENLPSLCSUQGLR^334^ ^335^ AEENITTESCQUR^346^	3 Isoforms: 45 kDa (terminated at 299) 49 kDa (*n.d.*) 57 kDa (*n.d.*)	[65]
Human breast milk	^312^TGSAITUQCKENLPSLCSUQGLR^334^ ^370^NQAKKUEUPSNC^382^	*n.d.*	[83]
Human serum	^299^SUCCHCR^305^ ^311^TGSAITUQCK^321^ ^322^ENLPSLCSUQGLR^334^ ^335^AEENITESCQUR^346^ ^347^LPPAAUQISQQLIPTEASASUR^368^ ^368^UKNQAK^374^ 375KUEUPSN^381^	*n.d.*	[90]
Human serum	^38^DQDPMLNSNGSVTVVALLQASUYLCILQASK^68^ ^311^TGSAITUQCK^321^ ^322^ENLPSLCSUQGLR^334^ ^335^AEENITESCQUR^346^ ^347^LPPAAUQISQQLIPTEASASUR^368^	*n.d.*	[91]

*n.d.*: no data.

## 5. Quantification of SELENOP

### 5.1. Immunoassays

Originally, radioimmunoassays based on the in-vitro synthesized or partially purified labeled SELENOP preparations were proposed by [19,63,92,93]. Nevertheless, due to the cumbersome procedures and restrictive regulations concerning the use of radioactive materials, these assays were only used in very few studies and were not widely adopted by the larger community.

ELISA (enzyme-linked immunosorbent assay) for SELENOP was first developed as early as 2010 [24], but the early ELISA assays were time consuming and inconvenient for clinical use. However, the approach is rapidly gaining popularity for the absolute quantification of SelP in plasma samples [59,94]. Several kits are commercially available [73,74,95,96,97]. The results depend largely on the kit used and caution and criticism are required when comparing data obtained with the different kits [98].

The accuracy of the results depends on the epitope identified by the antibody and its selectivity (activity towards the other proteins). If an antibody recognizes just, for example, the N-terminal, the assays will capture not only full-length SELENOP but also the SELENOP-N-terminal fragment [52]. An assay using colloidal gold particles coated with two types of anti-SELENOP monoclonal antibodies, one recognizing the N-terminal side domain and the other recognizing the C-terminal, was developed for measuring full-length selenoprotein P in human serum [52].

The in-plate variation, within-laboratory variation, and between laboratory variation are all typically below 15% with a limit of quantification of 10 ng/mL [59]. The accuracy of ELISA (difference with the SRM value) was assessed to be 2.9% [59]. Because of the limited presence of mass spectrometry technology in the clinical environment, immunoassays are the most widely used technique for human and animal health status.

### 5.2. Liquid Chromatography—Inductively Coupled Plasma—Mass Spectrometry(ICP-MS)

SELENOProtein P can be quantified by measuring the Se response provided that the protein is separated from the other Se-containing species by HPLC. The initial use of hydride generation atomic absorption or fluorescence spectrometry was replaced by ICP-MS because of its higher sensitivity, isotopic specificity and simplicity avoiding the need for post-column chemical conversion of SELENOP to Se or to selenium hydride. Because of the high relative abundance of SELENOP, the challenge of separation is practically limited to SELENOP from selenoalbumin, GPx3, and selenometabolite fraction.

**Figure 3:**
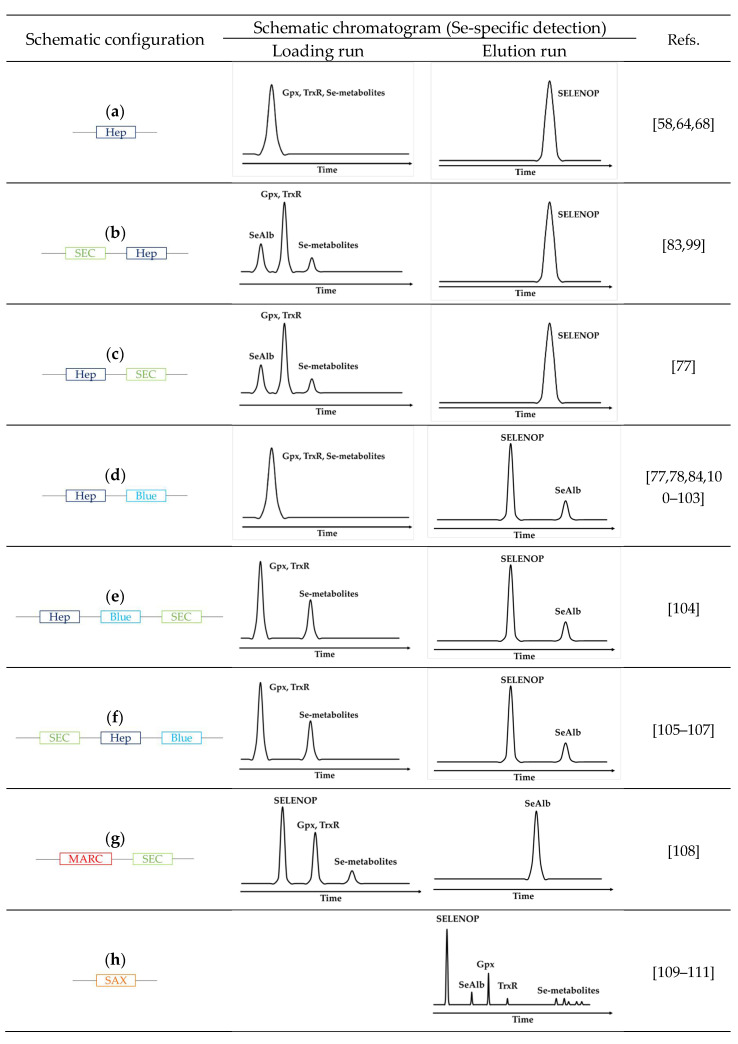
Schematic overview of the principle of HPLC configuration coupled with ICP-MS for SELENOP determination (**a**) schematic chromatogram of affinity column heparin coupled with ICP-MS (Se-specific detection) [58,64,68], (**b**) schematic chromatogram of a size exclusion column followed by an affinity column Heparin coupled with ICP-MS (Se-specific detection) [83,99], (**c**) schematic chromatogram of affinity column heparin followed by a size exclusion column coupled with ICP-MS (Se-specific detection) [77], (**d**) schematic chromatogram of multi-affinity columns heparin followed by blue-sepharose coupled with ICP-MS (Se-specific detection) [77,78,84,100,101,102,103], (**e**) schematic chromatogram of multi-affinity columns heparin followed by blue-sepharose and a size exclusion column coupled with ICP-MS (Se-specific detection) [104], (**f**) schematic chromatogram of a size exclusion column followed by multi-affinity columns heparin and blue-sepharose coupled with ICP-MS (Se-specific detection) [105,106,107], (**g**) schematic chromatogram of multi-affinity removal column followed by size exclusion column coupled with ICP-MS (Se-specific detection) [108], (**h**) schematic chromatogram of a strong anion-exchange column coupled with ICP-MS (Se-specific detection) [109,110,111], (Hep: HiTrap Heparin affinity column, SEC: Size-exclusion chromatography, Blue: HiTrap Blue affinity column, MARC: Multi-affinity removal column, SAX: Strong anion-exchange column).

The principle of the methods is based on downscaling and on-line arrangement in different configurations of the three principal techniques discussed above: size-exclusion LC for the separation according to the molecular weight, heparin LC for the selective retention of SELENOP, and Blue-Sepharose column for the selective retention of SeAlb. As a result, the SELENOP signal is obtained in the form of a chromatographic peak, other species may sometimes be separated by SEC. The principle of the proposed arrangements and the type of signal used for quantification is schematically shown in (Figure 3).

The most widely used principle for the on-line isolation of SELENOP consists of the retention of SELENOP by affinity using a heparin column while all other selenium species are eluted and detected as a peak in the loading run [68]. The subsequent elution run produces a peak corresponding to SelP (Figure 3a). The incorporation of a SEC (heparine) column on-line, either preceding [83,99] (Figure 3b) or following [77] (Figure 3c), allows the discrimination of the selenium species eluting in the loading run into HMW Se-containing proteins (Se-albumin and GPx) and LMW metabolite fraction. 

A more sophisticated version of the system includes a switching valve and a circuit containing a column with an affinity for albumin, allowing for the specific recovery of SelAlb during the loading run for its subsequent quantification (Figure 3d) [84,100,101,102,103]. It can be refined by the integration of a SEC column into the system (Figure 3e–f) [104,105,106,107].

Size-exclusion LC alone does not offer a sufficient resolution nor preconcentration to separate SELENOP form SelAlb [112,113]. However, when coupled in-tandem with a SelAlb method, SELENOP appears as a peak partially separated from GPx3 and separated from LMW selenometabolite fraction (Figure 3g) [108].

Alternatively, as SelP is the only selenoium species in serum and cerebrospinal fluid not retained on the anion-exchange column; anion-exchange HPLC (Figure 3h) was used to separate it from other species [109,110,111].

#### 5.2.1. Selectivity and Sensitivity ICP-MS Detection 

ICP-MS allows a specific detection of the individual selenium isotopes. Selenoprotein P can be therefore quantified via its selenium content once it has been separated from the other selenium-containing species. Even if the sensitivity of ICP-MS for selenium is inferior to that for most metals because of its higher first ionization potential (9.75 eV), higher proneness to matrix suppression, and the split of ions available among six isotopes, it is fully compatible with the requirements for SELENOP detection in HPLC. 

The main problem is the choice of the least interfered isotope as all the Se isotopes can be interfered by polyatomic ions: Ar_2_ dimers or Ar combinations with Br, S, or Br. A possible interference is ^156^Gd in serum of patients having received Gd contrast agents were also evoked [68]. The interfering elements can be separated by HPLC [102] and are usually not a problem which favors the choice of ^77^Se (7.63% abundance) or ^82^Se (8.73%). The interference on ^78^Se^+^ can be effectively eliminated by the use of a H_2_ pressurized collision cell [114]. The use of the most abundant ^80^Se (49.6%) requires the removal of the Ar_2_^+^ ions which can be achieved by using a collision cell installed between two quadrupoles as it is the case in the ICP-MS/MS instruments [114]. The latter also allows the non-interfered detection of the ^80^Se^16^O^+^ ion produced by the oxidation of Se in the reaction cell [114]. 

#### 5.2.2. Calibration and Quantification 

A major problem in the quantification of SELENOP is the non-availability of an authentic SELENOP standard as it is currently practically impossible to obtain recombinant SELENOP in reasonable purity and quantity. Calibration is therefore carried out using a proxy such as selenite, selenomethionine, a peptide characteristic of SELENOP, or recombinant homologue of SELENOP in which the Sec residues were replaced by Cys. 

The standards used for calibration are usually isotopically labeled. However, the term “isotope dilution analysis (IDA)” frequently used in the context of quantification of SELENOP by HPLC-ICP-MS does not bear the original meaning of absolute (traceable to SI units) quantification. As there is not isotopically labeled SELENOP available, the analyte (SELENOP) and the spike used are not in the same chemical form.

The principal quantification strategies of SELENOP using calibration by isotope dilution are based on:

1. the measurement, in an HPLC peak, of the intensity ratio between the natural selenium isotope from the SELENOP after HPLC separation of the latter and the enriched Se (^77^Se, ^74^Se) added post-column as SeMet or selenite (“spike”). The method is referred to as species-unspecific isotope dilution [83,84,101,104,105,107].
2. the measurement, in an HPLC peak, of the intensity ratio between Se in selenomethionine obtained by the complete proteolysis of the selenoprotein and the isotopically enriched ^77^SeMet standard (“spike”). The method can be referred as species-specific isotope dilution on the amino-acid level. The proof of principle was demonstrated by Ruiz Encinar et al. [115] and the approach was applied to the SELENOP quantification by Jitaru et al. [116].
3. the measurement, in an HPLC peak, of the intensity ratio between the Se in a Se-containing peptide obtained from a selenoprotein by tryptic digestion and the isotopically enriched Se (^77^Se, ^74^Se) in the identical synthetic peptide. The addition of peptide spikes to the plasma samples was followed by tryptic digestion, alkylation, and isotope ration determination using HPLC-ICP-MS. The principle of the method was demonstrated by Polatajko et al. [117]. The method was first applied to the quantification of SELENOP by Ballihaut et al. [84]. Deitrich et al. [53] proposed the use of two synthetic peptides (isotopically enriched, Se^76^) derived from SELENOP for its quantification by IDA-ICP-MS/MS. The disadvantage is the introduction of additional uncertainty due to the necessity of control of the efficiency of the enzymatic digestion the use of SELENOPeptides [105].
4. the use of a proxy protein as standard. The closest proxy protein has been for the moment a full-length human recombinant SELENOP in which the original 10 SeCys residues (Sec) were replaced by 10 cysteines (Cys). This replacement was achieved by point mutations of nucleotide triplets coding Sec to triplets coding Cys in the coding sequence for selenoprotein present in the expression vector used as a template for E.coli protein synthesis [100]. Selenium was introduced in the polypetide chain during cell-free protein *E.coli* synthesis in the form of SeMet or ^76^Se-Met for the preparation of the SEPP1 standard and an isotopically labeled spike for isotope dilution analysis [118,119]. The standard and spike were purified by SDS PAGE [100]. However imperfect the assumption of the similarity of the behavior of the standard and the spike might be, the use of such an isotopically enriched spike allowed the quantification by standard addition and isotope dilution analysis by ICP-MS after the purification of SELENOP by affinity chromatography.

### 5.3. Gel-Electrophoresis-Based Methods

Isoelectric focusing (IEF) and sodium dodecyl sulphate-polyacrylamide gel-electrophoresis (SDS-PAGE) allow for the separation of full-length and truncated SELENOP forms. The quantification of SELENOP and its isoforms in the produced band(s) can be achieved by the detection of selenium (either radioactive or not) or directly by the detection of the protein (recognized by a specific antibody). 

The mature SELENOP has a molecular mass of 41 kDa but migrates as multiple bands, of approximately 50–60 kDa in SDS PAGE, probably due to variations in glycosylation (conserved 2 N-linked—and one O-linked glycosylation) [47]. Deglycosylation shifts the migration band from 57 kDa to 43 kDa [93]. A small difference can bee seen in mobility (69 kDa under nonreducing conditions) and 66 kDa under reducing conditions [79].

#### 5.3.1. ^75^Se Detection 

The migration of ^75^Se radioactivity in the gel played a fundamental role in the early works, allowing for the discovery and rough characterization of SELENOP [17,18]. The comparison of the radioactivity in the band with that of a standard is a straightforward method of quantification. An additional advantage is a convenient evaluation of SELENOP recovery from the gel. The inconvenience is the need for handling gamma-radioactivity which requires dedicated laboratories. Note that the technique requires that the quantified ^75^Se is present in the radioactive form. Whereas it is an elegant quantification technique in animal studies, it is not an option for human SELENOP.

#### 5.3.2. Western Blotting Detection

The principle of the method consists of the separation of SELENOP by SDS PAGE and its transfer onto a PVDF membrane. The membranes are subsequently incubated with primary and secondary antibodies and developed with enhanced chemiluminescence. Standard curves were constructed using a dilute NIST1950 reference material [49,96].

The method is particularly attractive in combination with immunoprecipitation [55,65] allowing the isolation and preconcentration of SELENOP from complex samples.

The selectivity of the method depends less critically on the quality of antibodies than in the case of ELISA as SDS PAGE offers an additional separation step. The method does not account for losses at the different stages of the procedure, but many of these losses are compensated by the calibration with a standard reference material. Western blotting is robust but is not traceable to the SELENOP sequence.

#### 5.3.3. Laser Ablation-ICP MS Detection 

**Figure 4:**
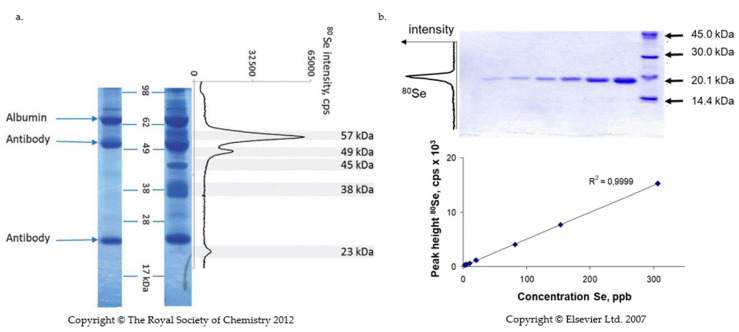
Quantification of SELENOP by laser ablation ICP MS. (**a**) SDS -PAGE of immunoprecipitated SELENOP from serum. Left lane: albumin standard and antibodies bands; Right lane: sample lane; graph: ^80^Se intensity as a function of position in the gel [65]; (**b**) Construction of a calibration curve in SDS PAGE—laser ablation ICP MS with GPx [121]. The calibration curve is a linear function of the quantity of protein in the gel. G. Ballihaut, L.E. Kilpatrick, E.L. Kilpatrick, W.C. Davis, Multiple forms of selenoprotein P in candidate human plasma standard reference material, Mettalomics, 2012, 4, 6, 533-538, with permission of Oxford University Press; Reprinted from TrAC Trend in Analytical Chemistry, 26, 3, 2007, G. Ballihaut, C. Pécheyran, S. Monicou, H. Preud’homme, R. Grimaud, R. Lobinski, G. Ballihaut, R. Grimaud, R. Lobinski, Multimode detection (LA-ICP-MS, MALDI-MS and nanoHPLC-ESI-MS²) in 1D and 2D gel-electrophoresis for selenium-containing proteins, 183-190, Copyright (2007), with permission from Elsevier.

The detection consists of the evaporation of SELENOP present in the band separated by isoelectric focusing (IEF) or SDS PAGE using a laser beam followed by the quantification of selenium in the produced aerosol by ICP-MS. This method was first proposed by Fan et al. [120] and later developed for the quantification of selenium proteins by Ballihaut et al. [121,122] and Bianga et al. [123]. The analytical signal resembles a chromatogram (Figure 4).

The identity and purity of the band can be verified by tryptic digestion and ESI MS/MS analysis of the non-ablated part of the gel. Calibration is carried out in a parallel lane using a well-characterized Se-containing protein with a known Se concentration, such as a fully selenized calmoduline [122] or glutathion peroxidase [121]. 

The LA-ICP-MS detection limits were reported to be 10 times lower for GPx than those of Western blot analyses [124]. However, because LA-ICP-MS is sensitive to selenium and the number of Se atoms in SELENOP is ten times bigger than in GPx, similar detection limits to those of Western blot are expected for SELENOP. Indeed, in well optimized conditions, the reported sensitivity was close to that of radioactivity detection [125]. Note that the advent of triple quadrupole ICP-MS offers a considerable margin for improvement of the detection limits as the most abundant ^80^Se can be chosen for detection [124]. LA-ICP-MS offers a dynamic range of five orders of magnitude which largely surpasses that of Western blotting detection (two orders of magnitude) and of ELISA (one order of magnitude) which is important for the analysis of unknown sample.

### 5.4. Isobaric Tagging for Relative and Absolute Quantification (i-TRAQ)

The use of LC-MS/MS as a way to quantify SELENOP was developed with the advent of new techniques such as label-free quantification (LFQ), and tandem mass tag [126].

The iTRAQ (isobaric tagging for relative and absolute quantification) method is based on the covalent labeling of the N-terminus and side chain amines of peptides from protein digestions with tags of varying mass. The samples are then pooled and liquid chromatography tandem mass spectrometry (MS/MS). A database search using the fragmentation data allows for the identification of the labeled peptides and hence the corresponding proteins. The fragmentation of the attached tag generates a low-molecular-mass reporter ion that can be used to relatively quantify the peptides and the proteins from which they originated [127].

To enhance the detection and identification of medium- and low-abundance proteins (such as SELENOP), different immuno-depletion methods are usually used. Since lipids interfere with iTRAQ labeling (as well as detection of peptides by mass spectrometry), the serum samples have to be delipidated before the analysis [80].

SELENOP has been reported in iTRAQ-based multiplex quantitative proteomics investigations searching for biomarkers of disease [80,87,88,89,128,129] or of Se-exposure [130].

### 5.5. Quality Control and Assurance 

As discussed above, several assays for the quantification of human SELENOP have been described. The reported serum concentrations of SELENOP differ considerably between assays, areas and type of diseases, as well as the laboratories. It is unclear whether the reason for this is biological variations or inadequacy of the analytical methodology [47]. Therefore, there is an urgent need for a better understanding of the sources of error in SELENOP analyses. 

The SELENOP instability in the sample can be a source of error regardless of the analytical technique used. SELENOP was reported to be stable in serum over prolonged periods of time and upon repeated freezing [68,94] but other researchers found it to decompose quickly and lose selenium [110]. SELENOP proteolysis upon storage and its interactions with abundant or specific plasma proteins may occur [23]. In order to reduce the risk of autolysis, diisopropyl fluorophosphate was recommended to be added to each pooled fraction to avoid the proteolytic cleavage of SELENOP [79]. The question of the stability of semi-purified SELENOP has not been widely discussed but the matrix removal is likely to affect the stability of the protein. 

Each of the techniques proposed has its own sources of error. Antibody-based enzyme immunoassays suffer from a difficult-to-control selectivity and high standard deviations. The validation of most commercial assays for SELENOP had been missing until 2015 [72] leading to inconsistencies and poor data comparability. The SELENOP quantification is closely linked to the choice of the kit and the quality of the antibody employed. A recent comparison of the commercially available kits for the determination of SELENOP levels in 21 human serum samples showed that only one out of the three commercial kits tested showed a good correlation with the method developed in-house [98]. 

In chromatographic methods, the peak assignment is based on the retention hypothesis which assumes the specificity of the separation to SELENOP. The molecular confirmation of the identity of the SELENOP determined (full length, truncated, post-translationally modified) is often lacking. The SELENOP recovery in the separation procedures is seldom reported. However, the traceability for the Se mass balance (100% column recovery), the unambiguous identification of the SELENOP eluted, and the verification of the fraction purity are the *sine qua non* conditions to assign the accurate value to the SELENOP concentration [84].

On the level of quantification by ICP-MS, calibration by unspecific or semi-specific isotope dilution does not account for any losses or transformations occurring during sample preparation or species separation. The assumption of the identical response of the selenite and Se present in SELENOP may not be true under some conditions. The precision of the isotope ratio measurement is a considerable source of measurement uncertainty at levels close to the detection limits [53] which requires improvements in terms of sensitivity.

The accurate quantification is contingent on the availability of specific certified reference materials (CRMs), pure primary standards and reference methods. Interlaboratory assays have been scarce and limited to a single type approach, e.g., to ELISA [98]. A comparison of Western blotting with ELISA [96] showed that the results from both methods were in agreement and indicated an unchanged pattern of immunoreactive protein isoforms.

A great step towards quality assurance is the increasing number of works reporting the SELENOP concentration values in the CRMs available on the market. The data reported by several authors are summarized in Table 2 which shows a good coherence of the results obtained by ICP-MS approaches. However, no comparison of this data obtained with immunological methods has been available, most of these methods actually use the CRM as calibration standard [59].

## 6. Imaging

In the absence of imaging studies using mass spectrometry, the imaging of SELENOP in tissues can be carried out using either chemical fluorescent probes targeting free SeCys residues or by immunohistochemistry using SELENOP directed antibodies. 

The use of the strong nucleophilic properties of selenols to simulate the cleavage of Se-Se and Se-S bonds is a widely used mechanism for designing the fluorescent probes for the detection of bioselenols validated for thioredoxine reductase. Currently, few selenol specific fluorescent probes are commercially available for medical diagnosis or SELENOP detection, and it is difficult to distinguish various functional selenoproteins by detecting selenol [132]. 

Antibodies can be used for selective imaging of SELENOP in tissue slices. The methods include: immunoperoxidase staining, immunofluorescence, and immunohistochemical analysis in paraffin-embedded samples. Immunohistochemical localization of SELENOP revealed its presence in liver and brain bound to capillary endothelial cell walls [49].

## 7. Applications and Reference Values

Table 3 summarizes the results of clinical studies in a variety of areas reporting SELENOP concentration in serum samples of healthy individuals and patients affected by specific diseases, measured by different molecular (immunoassays) or elemental methods (ICP). The variations in the data obtained by different techniques and laboratories are significant. In practice, clinical conclusions can be drawn solely on the basis of data obtained in the same laboratory.

Most of the studies have been limited to serum. A recent study of breast milk from five healthy mothers reported a SELENOP concentration of 20.1 + 1.0 ng/mL (as Se) by HPLC -ICP-MS [83]. An early study of the cerebrospinal fluid of 24 neurologically healthy subjects by HPLC-ICP-MS resulted in a mean concentration of 0.47 ng/mL, a factor of 10 lower than the corresponding concentration in serum [110]. Recently, a study of 75 Parkinson’s disease patients reported a mean value of 1.92 ng SELENOP (as Se) /mL in comparison with 2.02 ng/mL found in the population of 68 control subjects.

The isotopic selectivity of ICP-MS and the fact that selenium has multiple isotopes offers a possibility to use stable isotopic tracers to follow the incorporation of Se coming from different food sources into selenoproteins, including SELENOP. The intravenously injected ^82^Se-enriched SeMet into mice under different nutritional statuses (Se-adequate and Se-deficient) before the isotope-specific measurements of the expressed selenoproteins were taken, allowed studies to discriminate part of the exogenous ^82^Se incorporated into SELENOP [113].

**Table 3:** Overview of SELENOP concentrations in blood sample studies involving several subjects. The values that can be consider as control are highlighted in grey.

Patients’ Characteristics (Number)	Country	SELENOP Average (Range), ng Se/mL		Technique Used for Quantification	Ref.
Healthy (73)	Japan	96.9 ± 20.1 *		ELISA (in-house kit)	[70]
Hypercolesterolaemic before treatment (7)		104 ± 20.1 *			
Hypercolesterolaemic after treatment (7)		42.1 ± 23.8 *			
Healthy (5)	Spain	54.8 ± 3.99		HPLC-ICP-MS (post-column IDA)	[101]
Haemodialysis (5)		35.2 ± 2.66			
Healthy (20)	Japan	62.2 ± 14.6 *		AAS	[133]
Ulcerative colitis (34)		54.8 ± 18.3 *			
Crohn’s Disease with elemental diet (17)		32.9 ± 9.14 *	25.6 ± 7.32 *		
Crohn’s Disease with non-elemental diet (20)			38.4 ± 5.49 *		
Healthy (318)	Germany	98.4 ± *n.a.*		ILMA (in-house kit)	[94]
Healthy (399)	Greece	49 ± 15		HPLC-ICP-MS (post-column IDA)	[104]
Healthy (15)	Italy	56 ± 8		HPLC-ICP-MS (external calibration)	[103]
Type 2 diabetes (40)		58 ± 9			
Healthy (20)	Korea	6.62 (4.62–12.7) ± *n.a.* *		ELISA kit USCN Life Science	[97]
Type 2 diabete (40)		18.9 (9.07–39.3) ± *n.a.* *			
Prediabete (40)		15.9 (9.44–28.9) ± *n.a.* *			
Neurologically healthy (24)	Germany	1.55–50.6 ± 0.03		HPLC-ICP-MS (external calibration)	[110]
Control (966)	Europe	78.6 (53–112) ± 16.9 *		ILMA Selenotest^TM^, ICI GmbH	[38]
Patient with colon cancer (598)		75 (49.4–110) ± 16.9 *			
Patient with rectal cancer (368)		76.8 (53–110) ± 16.9 *			
Healthy mother (83)	Spain	42,49 ± 9.49 (ICP-MS)		HPLC-ICP-MS (post-column IDA) Elisa kit USCN Business Co	[107]
		6.99 ± 2.26 (ELISA) *			
Healthy baby (83)		28.06 ± 7.69 (ICP-MS)			
		0.35 ± 0.18 (ELISA) *			
Healthy (29)	China	265 ± 234 *		ELISA kit USCN Life Science	[95]
Overweight/obese individuals (34)		957 ± 715 *			
Healthy (76)	Japan	45.9 ± 9.51 *		Sol particle homogeneous immunoassay (SPIA)	[66]
Occupationally non-exposed (50)	Germany	31.1–59.2 ± (2.18–4.14)		HPLC-ICP-MS (external calibration)	[68]
after coronary angiography (controls) (20)	Japan	44.4 ± 4.94 *		sol particle homogeneous immunoassay (SPIA)	[27]
pulmonary arterial hypertension patients (65)		56.1 ± 10.4 *			
Control (around 2000)	Sweden	80.5 ± *n.a.* *		ELISA Kit selenOtestTM, SelenOmed GmbH (validated by Western blot)	[96]
Patient before selenium treatment (55)		62.2 ± *n.a.* *			
Patient treated with 0.5 mg selenite/m²		73.2–128 ± *n.a.* *			
Patient treated with 1–33.4 mg selenite/m^2^		>183 ± *n.a.* *			
Control group (37)	Germany	67.7 ± *n.a.* *		ELISA kit Cloud Clone	[134]
infarct-related cardiogenic shock day 1 (147)		112 ± *n.a.* *			
infarct-related cardiogenic shock day 3 (147)		411 ± *n.a.* *			
Healthy (39)	Spain	76.74 ± 3.72		HPLC-ICP-MS (SUID)	[105]
Lung cancer (48)		82.04 ± 4.41			
Control group (966 EPIC study)	Germany	78.6 (53–112) ± 16.9 *		ELISA Kit selenOtestTM, selenOmed GmbH	[135]
Liver transplanted patient alived (63)		37.9 (11.7–85.2) ± 12.8 *			
Liver transplanted patient deceased (16)		36.2 (18.5–60.7) ± 11 *			
Controls group (1160)	Denmark	101 (64–146) ± *n.a.* *		CE-certified SELENOP-ELISA	[136]
Advanced prostate cancer patient (1160)		98.8 (62.2–146) ± *n.a.* *			
High-grade prostate cancer patient (1160)		101 (65.8–146) ± *n.a.* *			
Advanced-stage prostate cancer patient (281)		98.8 (58.5–148) ± *n.a.* *			
Type 2 diabetes patients (176)	China	33.2 ± 8.93 *		ELISA kit Cloud Clone	[42]
Healthy mother (20)	Spain	57.1 ± 8.7 (ICP)		HPLC-ICP-MS (SUID) SELENOP-ELISA Kit	[137]
		22.1 ± 10.6 (ELISA)			
Healthy baby (20)		33.6 ± 4.2 (ICP)			
		11.8 ± 2.8 (ELISA)			

* Values have been recalculated to be expressed as concentration of Se (ng/mL) in SELENOP, assuming that SELENOP contains 10 atoms of Se in its sequence and its molecular weight of 43,174 g·mol^−1^. *n.a.* = non-available.

## 8. Conclusions

SELENOP holds promise to be a valuable biomarker of selenium status, but its chemical characteristics still need to be investigated and methods for its accurate quantitation improved.

Immunoassays remain the standard for the determination of human and animal health status, because of their speed, simplicity and limited presence of mass spectrometry technology in the clinical environment. The validation of assays has been considerably improving and interlaboratory comparisons undertaken in order to prevent erroneous data and incorrect interpretations. In particular, critical comparisons of data obtained using methods based on a different principle: immunoassays and mass spectrometry are necessary. Before recombinant SELENOP becomes available to be used as a primary standard for isotope dilution quantifications, careful investigation of the characteristics of the SELENOP measured by electrospray MS and strict control of the recoveries at the various steps of the analytical procedures are strongly recommended.

The molecular characterization of SELENOP isolated from different species is required to address the questions of truncated isoforms; those varying in terms of amino-acid composition and sequence, as well as in terms of post-translational modifications require more in-depth studies which would definitely profit from the approaches of top-down proteomics. To date, the results yielded by the classical protein analysis, peptide mapping and microsequencing, remain fundamental to our knowledge about the isoforms. MALDI FT-ICR imaging is indispensable to verify the imaging data obtained by selenol probes and immunostaining of tissue slices. 

While most studies into SELENOP’s characterization have been carried out on rats, the exploration of the SELENOP expression in organisms theoretically capable of incorporating dozens of SeCys residues into a SELENOP sequence is a fascinating topic [11].

## Figures and Tables

**Table 2:** SELENOP concentration values reported for the analysis of standard reference materials by HPLC-ICP-MS.

Certified Reference Material	Calibration (Calibrant)	SELENOP Average Concentration (ng/mL as Se)	Ref.
SRM 1950	On-line post-column IDA (^77^Se)	105 ±3.8	[84]
	IDA (^76^ Se enriched peptides)	60.6 ± 3.2	[53]
	On-line post-column IDA (^77^Se)	61.1 ± 7.4	[100]
	External (SelP)	52 ± 1.6	[68]
BCR 637	External (selenite)	51 ± 1	[102]
	On-line post-column IDA (^77^Se)	52 ± 5	[104]
	On-line post-column IDA (^77^Se)	81 ± 7	[84]
	On-line post-column IDA (^77^Se)	52.7 ± 4.3	[100]
	IDA (^76^Se enriched peptides)	56.2 ± 1.7	[53]
	On-line post-column IDA (^74^Se)	61 ± 4.3	[105]
BCR 638	On-line post-column IDA-ICP-MS (^77^Se)	65 ± 5	[131]
	External (selenite)	57± 2	
	External (selenite)	59± 1	
	On-line post-column IDA (^77^Se)	54 ± 12	[104]
BCR 639	External (selenite)	56 ± 1	[102]
	On-line post-column IDA (^77^Se)	52 ± 3	[104]

## Data Availability

Not applicable.

## Abbreviations

AA	amino-acid
AAS	atomic absorption spectroscopy
Alb	albumin
Blue	HiTrap blue affinity column
CRM	certified reference material
ELISA	enzyme-linked immunosorbent assay
ESI	electrospray ionizarion
FT-ICR	fourier-transform ion cyclotron resonance
Glu-C	endoproteinase glu-c = glutamyl endopeptidase
Gpx	glutathione peroxidase
Hep	HiTrap heparin affinity column
His	histidine
HMW	high molecular weight
HPLC	high pressure liquid chromatography
ICP-MS	inductively coupled plasma- mass spectrometry
IDA	isotopic dilution analysis
IEF	isoelectric focusing
ILMA	immunoluminometric assay
IMAC	immobilized metal ion affinity chromatography
iTRAQ	isobaric tagging for relative and absolute quantification
LA	laser ablation
LC	liquid chromatography
LFQ	label-free quantification
LMW	low molecular weight
MARC	multi-affinity removal column
MALDI	matrix-assisted laser desorption/ionization
MS	mass spectrometry
NTA	nitrilotriacetic acid
PNGaseF	peptide: *N*-glycosidase F
PVDF	polyvinylidene fluoride
SAX	strong anion-exchange column
SDS-PAGE	sodium dodecyl sulphate- polyacrylamide gel-electrophoresis
SEC	size-exclusion chromatography
SECIS	selenocysteine insertion structure
SeCys	selenocysteine
SELENOP	selenoprotein P
SPIA	sol particle homogeneous immunoassay
SUID	species-unspecific isotopic dilution analysis
